# Trajectories of Idea Emergence in Dialogic Collaborative Problem Solving: Toward a Complex Dynamic Systems Perspective

**DOI:** 10.3389/fpsyg.2021.735534

**Published:** 2021-12-17

**Authors:** Liru Hu, Gaowei Chen

**Affiliations:** Faculty of Education, The University of Hong Kong, Hong Kong, Hong Kong SAR, China

**Keywords:** complex dynamic systems, attractor, dialogic collaborative problem solving, idea emergence, dialogism

## Abstract

According to the complex dynamic systems (CDS) perspective, learning emerges at various system levels. This study built a coherent theoretical framework based on CDS and Bakhtinian dialogic theory and further employed the concept of *attractor* (i.e., certain stable states that recur over time) in CDS theory to investigate the trajectories of idea emergence and how they diversified group outcomes in dialogic collaborative problem solving (D-CPS). Two contrasting groups were compared using visual and qualitative analysis approaches. The analysis based on idea tree diagrams showed that new ideas emergent in group discussion tended to attract local utterances and performed features of attractors in CDS in both high-performing and low-performing groups. The analysis based on idea hierarchy diagrams revealed how ideas emerged at various system levels. It was also found that status problems were likely to affect the functioning of regulative feedback loops, which might give rise to different structures of idea evolution. This study proposed CDS theory as an alternative perspective, augmented by the ethical considerations of Bakhtinian dialogism, for examining the dynamics of D-CPS.

## Introduction

In recent decades, many theories and frameworks have been developed to describe and explain peer collaboration processes, such as information processing, constructivism, social constructivism/sociocultural theory, and group cognition (Borge and Mercier, 2019). However, researchers have faced increased tensions when adopting these theories and frameworks to harmonize interpretative and computational methodologies and to understand the complexity of collaborative processes (Wise and Schwarz, 2017). In response to these theoretical concerns, some researchers have taken an interest in complexity theories to ease the conflicts among existing learning theories (Jacobson et al., 2016), complement existing theories on learning interactions (Zuiker et al., 2016), and break through limitations of existing mono-ecological approaches by focusing on *trans*-ecological disruptions (Borge and Mercier, 2019). Some researchers have brought in complexity theories to help align theories and methods in education research (Hilpert and Marchand, 2018), given that some studies in education hold a non-linear assumption about the nature of learning but still adopt traditional linear statistical techniques.

### Complex Dynamic Systems

The word “dynamic” in complex dynamic systems (CDS) emphasizes the temporal change occurring in this type of system, while “complex” describes the non-linearity and unpredictability of such a dynamic process. A CDS can be visually described as the temporal change in a ball rolling over an undulating landscape (Hollenstein, 2013). This system changes all the time among many possible states, with some being stable and recurrent and others being rare. The undulating landscape illustrates the multistable state space of a dynamic system.

The CDS ontology comprises several essential assumptions including hierarchy, dynamics, and emergence (Hilpert and Marchand, 2018; Kaplan and Garner, 2020; Koopmans, 2020). CDS interprets phenomena in terms of hierarchical diversity, constituting elements as subsystems nested in a larger system. The phenomena continuously change and spontaneously form a complex evolving and self-regulatory control mechanism. High-level novel behaviors emerge in the constant feedback loops within the system. The complex interaction among components leads to the irreducibility of a system to its components, which is a defining characteristic of complexity and distinguishes CDS thinking from other paradigms (Thietard and Forgues, 2011).

There is order underlying irregular behavior in complex systems (Picano et al., 2019). Although CDS are apparently random and aperiodic, they tend to settle into certain stable states over time and thus show regular changes or patterns of behavior. These stable states to which a system tends to gravitate are named *attractors* (Van Geert, 2003; Guastello and Liebovitch, 2009). Attractors determine the order underlying complexity (Boeing, 2016). Closer to the attractors, the system becomes more stable and less likely to be perturbed. In the illustrative example of a status ball rolling over an undulating landscape, the ball tends to get trapped in holes, which are attractors. The present study focused on detecting attractors to understand the temporality of learning interactions, which has gained increased attention in the community of learning sciences (Mercer, 2008; Reimann, 2009; Knight et al., 2017).

### Learning Interactions as Complex Dynamic Systems

Much evidence exists to show that learning interactions have features of CDS (Arrow et al., 2000; Ramos-Villagrasa et al., 2018; Koopmans, 2020; Ricca et al., 2020). Research on collaborative communications has established that the interaction process is not temporally homogenous but dynamically shaped by historical and contextual factors (Schegloff, 2007; Kapur et al., 2008; Mercer, 2008; Wise and Chiu, 2011). Human interactions are situated in specific historical, institutional, and cultural contexts. They also usually emerge dynamically, rather than being planned.

Previous research has identified some attractors underlying learning interactions that help structure the dynamic and non-linear process of collective thinking. Kapur et al. studied the evolution of cognitive regimes during online collaborative problem solving (Voiklis et al., 2006; Kapur and Kinzer, 2007; Kapur et al., 2008, 2005). They coded an utterance as 1, 0, or −1, according to whether the utterance helped the group approach the problem’s solution, maintain *status quo*, or deviate from the solution according to the random walk model (Ross, 1996). Kapur et al. (2008) further calculated the aggregated impact of the utterances on collective convergence toward the goal. They consistently found that the trajectories of convergence of different groups, whether solving well-structured or ill-structured problems, rapidly flattened out after early interactional exchanges. They drew an analogy between such locking-in mechanisms and attractors in complex adaptive systems and emphasized the theoretical and methodological implications for a more temporal and emergent view of group dynamics.

The object of student discussion has also been identified as a potential attractor. Bloom (2001) found that the constant generation of new concepts/notions around the object of discussion increased the complexity of the discussion. In this process, arguments and counterarguments formed positive and negative feedback loops, further increasing the complexity of the discussion while maintaining a similar structure. Stahl (2010, 2016) also claimed that shared interactional resources (e.g., the task) coalesced different levels of group discussion (e.g., individual, group, community) and guided continuous co-attention. Salomon (1993) described the co-evolution of individual and distributed cognition as spiral-like and characterized by the dynamic interplay and progressive development of individual engagement and joint products.

The present study focused on how a CDS perspective could contribute to research on the temporality of dialogic collaborative problem solving (D-CPS), whereby students solve a problem collaboratively—mainly or wholly through dialogic interactions. Specifically, this study sought to build on previous work to examine whether emergent ideas in D-CPS operated as attractors and, if so, how these attractors emerged in a complex discussion process and diversified group outcomes.

## Theoretical Framework

### Dialogic Collaborative Problem Solving

“Dialogic” has been a popular word in the literature on education. It has been understood as a general term for high-quality education related to dialogue (Sfard, 2020) and does not necessarily refer to the Bakhtinian dialogic framework (Wegerif, 2013). According to the ontological assumptions of Bakhtinian dialogism, dialogue is where and how humans exist and develop (Bakhtin, 1986). Dialogic education concerns how to develop the freedom and responsibility of a dialogic self, how to transform original illusions that self and objects as separated in an external fixed reality, and how to empower the oppressed to name their own reality by expanding their consciousness as well as transforming their social reality (Wegerif, 2013). Hermans (2004) proposes a model of *dialogical self-*informed by Bakhtinian dialogic framework. He argues dialogical self as a spatial and temporal process of positioning that involves a variety of voices that may be competitive or cooperative.

Epistemologically, Bakhtin (1929/1984) views that there is no fixed and final knowledge or truth, but that meanings are internal to and only exist in dialogues where various voices interanimate. Therefore, he argues that genuine learning only happens in genuine dialogue and truth is just an emergence but not the end of a dialogic trip. Bakhtin (1929/1984) emphasizes the *equal rights* of consciousnesses in dialogic interaction and suggests that *dialogic* depends on whether various voices are of equal rights rather than whether superficially it is in a discursive form of dialogue.

The present study views dialogue as an important end of education itself, not simply as a medium for learning according to Bakhtinian dialogism. Genuine dialogue as defined by Bakhtin (1929/1984) involves a plurality of unique and opaque consciousnesses of equal rights. Accordingly, the present study defines D-CPS as a complex dynamic process whereby two or more consciousnesses, with equal rights and each with its own world, combine but are not merged into a unity in the process of solving a shared problem. Students’ verbal engagement is an essential end in D-CPS. Joint solutions emerge from and only exist in group dialogue such that group members open themselves to each other’s voices and augment their own in the process.

### Coalescence of Dialogism and Complex Dynamic Systems

A dialogic theory of education is ontologically consistent with a CDS perspective on education that views learning as “something that emerges,” rather than “something that is” (Jacobson et al., 2016, p. 212). Dialogism emphasizes the relational nature of dialogue (Koschmann, 1999). An utterance only makes sense in relation to utterances it responds to or evokes (Bakhtin, 1986). New ideas naturally pop up in the interanimation of multiple independent voices. A CDS perspective on education also emphasizes a shift from a component-dominant to an interaction-dominant research paradigm (Hilpert and Marchand, 2018). High-level novel behavior within a system arises from interactions of its elements or subsystems and cannot be reduced to characteristics of its elements (Goldstein, 1999).

Dialogism differs from the CDS perspective on education in terms of its underlying ethical considerations. CDS focuses on common features of dynamic systems across disciplines which are not limited to humanities. It is a positivist, objective, and scientific approach (Sawyer, 2005). In contrast, Bakhtinian dialogism emphasizes that genuine dialogue requires the equal rights of various voices (Bakhtin, 1929/1984). Such an emphasis on equity reflects the underlying ethical considerations of dialogism (Matusov et al., 2019), which are echoed by similar claims of multivocality in the humanities (Parker, 2006). Therefore, the present study adopted CDS concepts and tools to understand the dynamics of D-CPS and highlighted the necessity of examining the evolution of each component in the system, given the essential ethical considerations of dialogism.

### Dialogic Collaborative Problem Solving as a Complex Dynamic System

**FIGURE 1:**
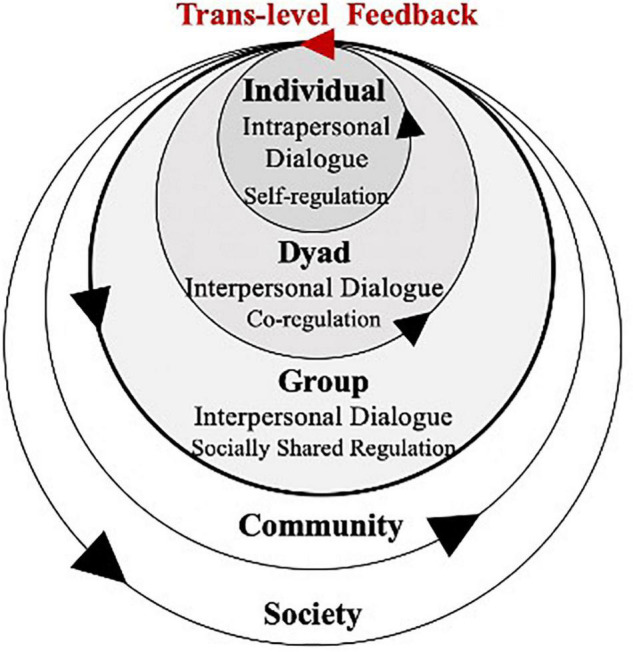
Dialogic collaborative problem solving as a complex dynamic system.

The present study illustrated a D-CPS system as a hierarchical structure embedded in a larger education system, which is visually analogous to Bronfenbrenner (1986) ecological systems theory (see Figure 1). According to Bakhtinian dialogism, genuine dialogue drives the flow of voices among co-equal consciousnesses. Therefore, the D-CPS system dynamically changes and evolves during both interpersonal and intrapersonal dialogue. Genuine dialogue forms constant feedback loops within and between different scales denoted by arrows. Truth or knowledge emerges in the dialogic interaction process and is therefore contextual, eventful, and never finalizable.

Stahl (2016) suggested that the “whole” in group work depended on whether it produced a unitary cognitive process, regardless of the number of people involved. As shown in Figure 1, the structure of a D-CPS system includes a dyadic layer that is unlike a three-layer (individual/group/community) micro-level structure, in that any dyad in a group is the smallest possible interaction subject and is able to generate dyad-level novel behaviors.

Social and social-interactive nature of human mind and behavior has been highlighted by concepts like transactive memory (Wegner, 1986), interactive minds (Baltes and Standinger, 1996), and group cognition (Stahl, 2010, 2016). Interpersonal dialogue, which may include challenging others’ contributions, inviting others to explain, or reflecting on joint performance, happens at both group and dyad levels in D-CPS. Dyad dialogue changes and evolves through co-regulations between pairs. Co-regulation refers to a transitional process through which individuals appropriate self-regulated learning through interactions with supportive others (Hadwin and Oshige, 2011). Meanwhile, group dialogue changes and evolves through socially shared regulations. Socially shared regulation describes how multiple others regulate their collective activity to build a joint understanding of the task, co-construct their goals and plans, and reach their common target (Järvelä and Hadwin, 2013).

According to Bakhtin (1929/1984), dialogue refers to a plurality of multiple independent voices but not the superficial discursive form. Therefore, an individual also engages in dialogue when they make meaning. The present study named this type of dialogue “intrapersonal dialogue” to distinguish it from “interpersonal dialogue.” Intrapersonal dialogue takes various forms, such as self-explaining, self-elaboration, or self-reflection. It evolves under self-regulation, which is the planning, monitoring, control or regulation, and reflection process of individual learning (Pintrich, 2000).

There are also *trans*-level feedback loops that give rise to self-organization and the emergence of the whole D-CPS system. Group dialogue is sustained through dynamic *trans*-level feedback among interpersonal and intrapersonal dialogues. In genuine dialogue, one individual adapts the other’s internally persuasive voice to his or her “own semantic and expressive intention” (Bakhtin, 1981, p. 293) and eventually appropriates the new voice. Empirical studies have also found that individuals were able to learn new problem-solving strategies from their peers during exploratory talk (Littleton and Mercer, 2013). Thus, individuals’ voices are changed and evolve through engagement with higher-level systems. In turn, an individual can help regulate dialogue, not only with another peer (i.e., co-regulation), but also with the whole group (i.e., socially shared regulation) and populate their voices to high-level systems. In this circular causality approach, individuals, dyads, and groups are all transformed, and novel properties emerge for dyads and the group.

In brief, both within-level and *trans*-level feedback loops drive and regulate the dynamics at various levels. Such feedback loops help the system and its substates move toward attractors, certain stable states in the D-CPS system. Therefore, identifying attractors and examining how within- and *trans*-level feedback loops move around these attractors is essential to understanding the dynamics and emergence of D-CPS.

**FIGURE 2:**
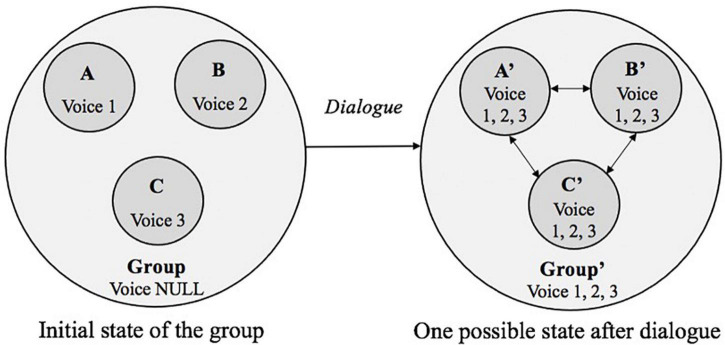
State change of D-CPS through dialogue (Note. Arrows indicate the direction of voices appropriation).

To further elaborate the dynamics of D-CPS, a three-person group is taken as an example (see Figure 2). In the initial state, A, B, and C are three independent equals, and each has one unique voice for the problem that is going to be solved together: voice 1, voice 2, and voice 3, respectively. In the beginning, there is no voice in the group space due to the lack of interaction. After the dialogue, one possible state of the group is that each voice is equally shared, and the members sufficiently resolve the different voices. In this case, all three students get two additional voices. Each dyad and the whole group all have three shared voices, accordingly.

Overall, this study sought to build a coherent theoretical framework based on CDS and Bakhtinian dialogic theory. As illustrated by both Figures 1, 2, this framework rests on four essential theoretical and conceptual assumptions.

First, D-CPS is a CDS without a pre-determined trajectory and is unpredictable. Truth emerges in genuine dialogue. It is contextual, eventful, and never finalizable.

Second, genuine dialogue is the plurality of unique and opaque consciousnesses of equal rights. Two consciousnesses can engage in genuine dialogue only when they are commensurable, open-minded, and equally important.

Third, voices can flow among co-equal consciousnesses only through genuine dialogue in D-CPS. Voices shared among dyads establish dyad-level truth, while those shared among all of the equal members enter the group level and establish group-level truth.

Fourth, the D-CPS system changes and evolves through regulation and feedback loops within and across various levels of the system.

## Materials and Methods

### Participants

This study was conducted in a primary school in a third-tier city of China in 2019. It is part of a project on temporal patterns and visualizations of D-CPS. The participants were 168 fourth graders from five classes (41% females, 59% males; 8 to 12 years old, 10.50 years old on average). They were informed of the overall project background (i.e., to study their collaborative dialogue) and the major task of this study (i.e., to finish three challenging mathematical problems in class). All of the participants volunteered to participate. Those who were unwilling to do the task were assigned other regular individual tasks and asked to be quiet and not disturb the other students.

To examine whether new ideas perform features of attractors in CDS and how new ideas emerge and diversify group outcomes, this study adopted a qualitative case study approach. The case study method is advantageous in analyzing and interpreting the complexities and subtleties of learners’ behavior and thoughts in social situations (Stake, 1995; Flyvbjerg, 2006). It allows in-depth and holistic descriptions and understandings and thus is very suitable to answer “how” and “why” questions (Yin, 2013). Therefore, the qualitative case study method matches the purpose of the present study.

We selected prototypical cases following three principles. First, the groups should produce similar amount of discussion for the target task. This principle tried to exclude possible influence of student engagement on group outcome and highlight the impact of interaction pattern. Second, group compositions should be similar in terms of size, gender, knowledge and social coherence. This principle tried to rule out the impact of grouping on interaction dynamics as well as group outcome. Upon satisfying the above two principles as far as possible, we further selected groups with contrasting solution quality on the target task to examine how the trajectory of idea emergence might influence group performance.

We finally presented two representative groups to examine the dynamics and structure of emergent ideas during D-CPS. The two groups were approximately similar in terms of the intensity of group interaction, member demographics, recent math grades and overall willingness to collaborate but differed regarding group outcomes (see Table 1).

**TABLE 1:** Background information on selected study participants.

Group	Student (pseudonym)	Gender	Participation rate	Chinese grade^a^	Mathematics grade^a^	Mathematics enjoyment^b^	Mathematics self-concept^b^	Social anxiety^c^	Willingness to collaborate^d^
High-performing	Wang	F	35%	106	104	4.00	3.67	1.00	10.00
	Gu	F	16%	108	99	2.11	1.44	2.40	8.00
	Yao	M	22%	106	103	2.78	2.44	2.10	2.67
	Gan	M	27%	106	113	3.78	3.13	1.20	6.67
Low-performing	Li	F	32%	109	108	3.89	3.44	1.40	5.00
	Yan	F	15%	78	85	3.89	2.22	1.80	9.00
	Bao	M	25%	114	98.5	4.00	2.75	2.00	9.33
	Xiao	M	27%	103	111	4.00	3.44	1.20	7.67

*^a^The maximum score is 120.*

*^b^4-point Likert scale.*

*The maximum score is 4.*

*^c^3-point Likert scale.*

*The maximum score is 3.*

*^d^Students’ average willingness to collaborate with their group members.*

*The maximum score is 10.*

### Settings and Procedure

The participants were organized into groups of four in their own classroom without computers during regular school time. Gender and prior mathematics grades were balanced across groups to the best of the teachers’ abilities. After all groups settled, the participants were required to write down the names of their group members and report their willingness to collaborate with their assigned group members on a scale from 1 to 10, with 10 representing the highest degree of willingness.

The groups were then instructed to collaboratively solve three structured open-process mathematical problems within half an hour and told not to discuss with other groups or touch the recorder in the middle of their table. To facilitate identification of the speakers, group members were required to introduce themselves according to a structured format before solving the problem. During the test, the teachers or researcher did not moderate the group discussions, except to clarify the task instructions or maintain classroom discipline.

After the test, the students independently completed a questionnaire concerning their demographic information, mathematics learning enjoyment, mathematics self-concept, and social anxiety. Mathematics self-concept and mathematics learning enjoyment were measured using items adapted from the questionnaire of Trends in International Mathematics and Science Study (TIMSS) for fourth graders in Taiwan (Mullis and Martin, 2013), with students being asked to indicate their agreement with each statement on a 4-point Likert scale (1 = *strongly agree*, 2 = *somewhat agree*, 3 = *somewhat disagree*, and 4 = *strongly disagree*). Social anxiety was measured using the 10-item Chinese version of the Social Anxiety Scale for Children–Revised (La Greca and Stone, 1993). The students were asked to indicate the frequency of specific behaviors on a 3-point Likert scale (1 = *always do this*, 2 = *sometimes do this*, 3 = *never do this*). The measures had a relatively high internal reliability, as indicated by Cronbach’s alpha values for social anxiety (α = 0.835), mathematics enjoyment (α = 0.734), and mathematics self-concept (α = 0.882) (Tavakol and Dennick, 2011).

### Materials

The level of difficulty increased throughout the three problems (featuring *ice cream*, a *snake*, and a *bridge*). The *ice cream* (item ID: M041132) and *snake* (item ID: M051006) problems were adapted from the TIMSS survey conducted in 2015 (TIMSS and PIRLS International Study Center, 2015). The *bridge* problem was the most difficult one and was adapted from the Junior Mathematical Olympiad (Database of Mathematical Olympiad, n. d.).

The students did not have any prior instruction in similar mathematical problems or on how to collaboratively solve a problem. To promote collaborative peer talk, the problems did not require the students to follow an explicit routine, although they all had unique correct answers. At the same time, the students had to rely mainly on their reasoning ability, rather than on their specific content knowledge, to find solutions to the problems; this ensured that the students with high levels of prior knowledge were not at an advantage.

The present study focused on group discussion in the *ice cream* problem (see the Appendix for the translated English version). This item required the students to calculate the unit prices for one ice cream and one popsicle when two ice creams and four popsicles cost 22 yuan, and one ice cream and three popsicles cost 14 yuan. Fourth-grade primary students haven’t learnt the concept of unknowns or the knowledge on equation sets. Therefore, this item couldn’t be solved following routines and requires students to reason based on the given conditions.

### Data Analysis

Written solutions submitted by the groups were graded according to a standard scoring criterion that considered the correctness of the final solution first and then awarded partial credit for solution steps informed by group discussion audio recordings if the final answer was wrong.

Group discussions were transcribed by turns. Each turn was coded from two dimensions: whether the turn contained a new idea and the productive talk moves in it, if any. This study considered three types of productive talk moves in peer talk: *reasoning*, *evaluation*, and *invitation*, which were summarized based on existing discursive productive talk moves identified in various contexts (e.g., King, 2002; Lazonder et al., 2003; Teo and Daniel, 2007; Michaels et al., 2010; Webb et al., 2014; Hennessy et al., 2016; Gillies, 2017). The *reasoning* type included intra-thinking talk moves, such as “elaborate,” “justify,” “speculate,” and “reflect” on one’s own contributions, and collective reasoning talk moves, such as “add on,” “co-justify,” “co-speculate,” and “co-reflect” on others’ or collective contributions. The *evaluation* type included evaluative talk moves such as “agree,” “disagree,” and general “evaluate”; while the *invitation* type included talk moves that aimed to invite someone to express (“invite to express”), reason (“why”), “say more,” and evaluate (“agree or disagree”). The first author and one trained coder independently carried out the coding of these two groups and resolved all disagreements.

**FIGURE 3:**
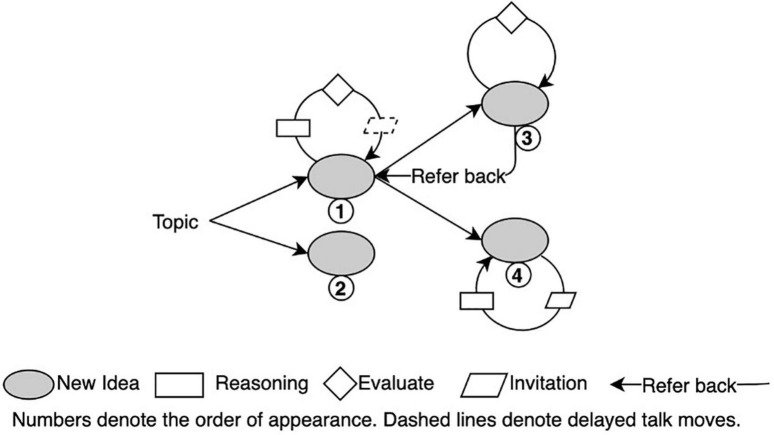
Structure of a possible idea tree.

This study further adopted two types of analytical diagrams to illustrate the cognitive trajectories of the group and help reveal possible attractors and multilevel interactions in D-CPS. The first, called an idea tree, depicts the knowledge evolution trajectory in the group space (see Figure 3). It numbers each new idea chronologically and illustrates productive talk moves around a certain idea through various shapes of boxes to show the local discussion on this idea. The idea tree also helps record the non-linearity of idea evolution. For example, students might refer back to previous ideas when they get stuck or realize mistakes. Such referring back is likely to generate new set of discussions around previous ideas. As shown in Figure 3, the idea tree uses reverse straight arrow to denote “referring back” and dashed boxes to denote productive talk moves around a previous idea.

The second was named an idea hierarchy, as shown in Figure 2, which was used to examine the state change of D-CPS. We used accumulated new ideas verbalized by individuals in D-CPS to represent the state of individual voices at that moment. Ideas shared at the lower level would emerge in the high-level space. The present study focused on the individual, dyadic, and group levels of D-CPS.

## Results

### Idea Emergence in the High-Performing Group

#### The Idea Tree Analysis

The 42 groups produced an average of 253 on-task turns (*SD* = 111) within half an hour, with a minimum of 101 turns and a maximum of 500 turns. For the selected high-performing group, 52 turns and 14 new ideas were produced in solving the ice cream problem.

**FIGURE 4:**
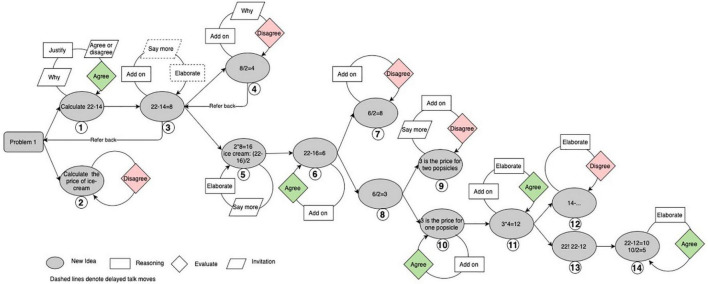
The structure of emergent ideas in the high-performing Group (Note. Talk moves classified as agree and disagree are colored. The ideas are numbered in the order in which they emerged).

The emergent new ideas formed an approximate binary tree structure for the high-performing group (see Figure 4). Utterances containing new ideas tended to attract several utterances that involved productive talk moves. Figure 4 visually represents that new ideas in this high-performing group performed features of attractors in CDS toward which local utterances tended to gravitate. Some ideas such as Idea 3 also attracted utterances beyond the local exchanges. For example, there was a “refer back” from Idea 4 to Idea 3. Idea 3 attracted a new round of discussions after the emergence of Idea 4 (i.e., talk moves denoted with dash lines) and further evolved into Idea 5.

The development of each new idea was largely determined by the regulative loop it went through. For example, at the very beginning, Gan proposed the first new idea: “calculate 22−14” at turn 30 (see Table 2). Then Wang pressed him for specific reasons at both turns 33 and 35. Gan explained that 22−14 would be equal to the price for one ice cream and one popsicle at turn 36. Next, Wang turned to Gu and asked her whether she agreed or disagreed with Gan at turn 37. Gu expressed agreement with Gan’s new idea and further proposed the third new idea by adding to Gan’s contribution at the following turn. Thus, the regulative loop involved in the development of the first new idea was “Why- > Justify- > Agree or disagree- > Agree.” This regulative loop involved co-regulated learning between Gan and Wang, self-regulated learning on the part of Gan induced by Wang’s questions, and socially shared regulated learning among Wang, Gan, and Gu. The agreement that was finally reached concerning the first new idea provided positive feedback on this thread of thinking and stimulated the development of the third idea and its further bifurcations of Ideas 4 and 5.

**TABLE 2:** Discussion excerpt of the high-performing group (translated from Chinese).

Turn	Speaker	Content	New Idea	Talk Move
27	Wang	Which one should we calculate first? I think we should first:		Invite to express
28	Gan	Let me have a look. Let me have a look!		
29	Wang	Come over and sit here. I think: I think: I think we should first calculate:		
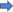 30	Gan	Calculate 22−14 first.	1	
31	Wang	Calculate the price for one ice cream first? (speak out together with Yao).	2	
32	Gan	22−14		
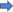 33	Wang	Why?		Why
34	Gan	22−14!		
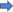 35	Wang	Why?!		Why
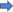 36	Gan	Because: this equals: the price for one ice cream and: and one popsicle.		Justify
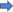 37	Wang	What do you think, Gu? (4.0)		Agree or disagree
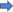 38	Gu	I think… I agree with Gan because they spent 22 Yuan and 14 Yuan in total, and the difference in their prices was 8 Yuan. And: and Ming has one more ice cream than Lin. Then:		Agree
39	Wang	Oh, wait a minute, wait a minute.		
40	Gu	Then, Ming has one more popsicle than Lin. Calculate 22−14 first: (3.0) equals to 8 Yuan. Then:	3	Add on
41	Wang	Yao, don’t do … anymore! (muffled sound, not clear)		
42	Gan	8 divides 2 equal 4	4	Add on
43	Gu	Why? Their prices are not necessarily the same.		Why Disagree
44	Gan	8 divides 2, equals 4. Listen to me (5.0), 8 yuan:		
45	Yao	Gan, I wanna ask a question… (muffled sound, not clear)		Say more
46	Gan	It means 8 equals one popsicle and one ice cream.		Elaborate

Figure 4 shows that positive feedback such as “agree” promoted the splitting of a certain idea branch and increasingly complexified group discussion. Meanwhile, “disagree” formed a type of negative feedback that tended to end the idea branch. For example, Gan proposed Idea 4, suggesting 8 divided by 2 directly at turn 42 (see Table 2). This was immediately challenged by Gu, who pressed him for his reasons and expressed disagreement, emphasizing that the unit prices for an ice cream and a popsicle could not be assumed to be the same. This negative feedback by Gu helped end the discussion of Idea 4 and further stimulated the emergence of Idea 5. Based on these multi-level and cross-level regulative feedback loops, individuals in this high-performing group constantly produced new ideas by elaborating on their own ideas or adding to others’ new ideas.

In brief, emergent new ideas in the high-performing group performed features of attractors in CDS. Emergent new ideas in this group mainly attracted local utterances and helped both diverge and converge group discussion.

#### The Idea Hierarchy Analysis

The trajectories of idea hierarchy were further illustrated to examine how dialogue drove the idea evolution and emergence at various levels of the system. In this study, we selected certain essential states to illustrate the evolution of idea emergence at various levels.

**FIGURE 5:**
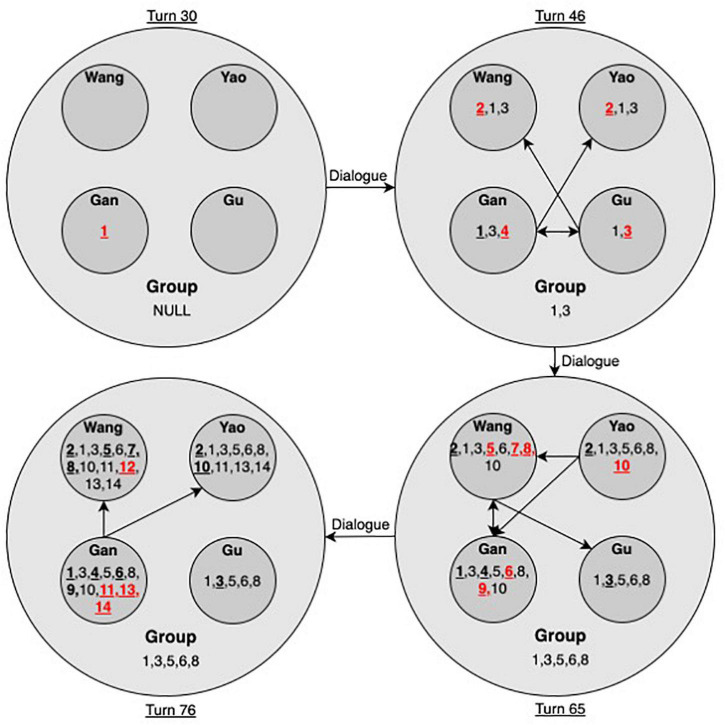
Idea hierarchy for the high-performing group (Note. Arrows denote the direction of idea flow. Numbers indicate the order of emergent ideas. Bold numbers with underlines indicate the idea was initially proposed by the indicated student. Numbers in red denote ideas that emerged in the current state).

In the initial state (turn 30), the first new idea emerged for Gan. Then, Wang and Yao proposed an alternative Idea 2 together. At turn 38, Gan’s idea was locally agreed to by Gu, who further added Idea 3. Wang seemed to give up her Idea 2 and accept Ideas 1 and 3, as indicated by her thinking at turn 39. Gan added Idea 4 to his Idea 3, which was immediately disagreed with by Gu. Therefore, Idea 4 failed to populate to the group. In addition, Gan helped Yao clarify the meaning of 8 at turn 46, which led to the sharing of Idea 3 by Yao as well. Until turn 46, the group was still stuck in Idea 3. Only two ideas (1 and 3) entered the group space (see Figure 5).

In the state at turn 65, the price for one popsicle was worked out (Idea 10). Idea 10 could be traced back to turn 51, where Wang put forward Idea 5. She worked out her idea with co-regulation by Gu, who challenged her calculation of 6/2 (idea 8). Idea 10 was shared by Wang, Yao, and Gan through interpersonal dialogue, while Gu did not vocally express her agreement with this idea. Therefore, until turn 65, the group had shared Ideas 1, 3, 5, and 9. In the final state, at turn 76, the price for the ice cream was also determined (Idea 14), which was mainly conducted by Gan with active participation of Wang. Yao expressed his understanding of the final answer at turn 74, while Gu did not vocally participate from turn 65 to 76. Therefore, the final idea was only explicitly shared by Wang, Gan, and Yao but not Gu.

In brief, the diagram of idea hierarchy, as shown in Figure 5, helped reveal the dynamics of idea emergence at the individual, dyadic, and group levels. New ideas attracted local utterances when they flew to other individuals and evolved through dialogue. In addition, idea hierarchy also helped reveal the dynamics of equity in D-CPS. Figure 5 indicates that Gan and Wang were the dominant contributors in the process, while Gu’s vocal participation was relatively less, especially during the latter phase of problem solving. Neither was the final answer vocally confirmed by Gu. As shown in Table 1, Gan and Wang also had the highest mathematics scores in the group, while Gu was the least competent in mathematics and had the lowest confidence. Yao had the equivalent level of mathematics grade as Wang but was not a prominent vocal contributor, perhaps because of his lowest level of willingness to collaborate with group members. Therefore, the dialogue of the high-performing group in solving the ice cream item was still constrained by certain status problems.

### Idea Emergence in the Low-Performing Group

#### The Idea Tree Analysis

**FIGURE 6:**
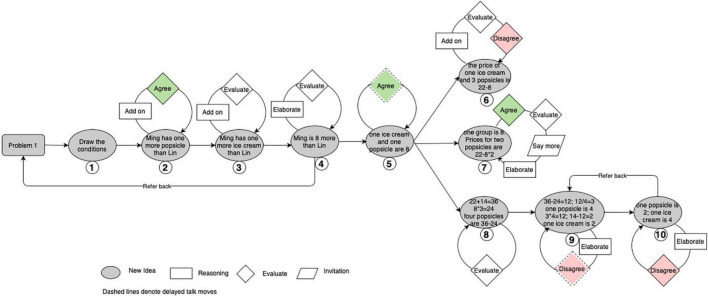
The structure of emergent ideas in the low-performing group (Note. Talk moves classified as agree and disagree are colored. The ideas are numbered in the order in which they emerged).

The low-performing group produced 67 turns and 10 new ideas in solving the ice cream problem. As in the high-performing group, emergent new ideas in this group also performed features of attractors in CDS (see Figure 6). In addition, there were some ideas that attracted utterances after another new idea (e.g., Ideas 5 and 9).

However, the evolution of the idea tree of the low-performing group was different from that of the high-performing one. There were fewer bifurcation points than in the high-performing group. Ideas evolved in a linear manner from Idea 1 to Idea 5, as shown in Figure 6. Participants in the low-performing group continued to put forward arguments until Idea 5, when they became a little stuck after calculating the total price for one popsicle and one ice cream. Then, the tree split into three branches at Idea 5.

The different evolution patterns of the idea trees across two groups were possibly because the regulative feedback loop in the low-performing group did not work as well as that in the high-performing one. For example, the positive feedback of “agree” on the seventh new idea did not help extend this idea branch. Instead, a new idea was generated from the fifth idea. As shown in the specific transcripts (see Table 3), it was Bao who first proposed the fifth idea at turn 61, which was taken up by Li at turn 67 and Xiao at turn 72. Li then added Idea 6 to Idea 5 at turn 69, in which she suggested calculating the price for one ice cream and three popsicles. Xiao disagreed with her and added Idea 7 to Idea 5 at turn 72, in which he suggested that one popsicle and one ice cream could be viewed as one group and then the price for two popsicles could be calculated from the money of Ming minus the money of two such groups. Idea 7 actually gave the correct procedure for working out the price for one popsicle, and Li immediately agreed with it, and again in turn 73. However, Bao seemed consistently unable to follow their discussion. He commented that the discussion between Li and Xiao did not make sense to him (turns 70 and 75). However, Li’s response to turn 70 did not help him clear his confusion, while his repeated comment at turn 75 received no response from either Li or Xiao. Turn 75 indicated that Bao’s negative comment was mainly addressed toward Li’s idea but not the correct idea by Xiao. This was possibly why Bao failed to build on Xiao’s Idea 7 but proposed Idea 8 (turn 98) when the group became stuck, which was directly based on his previous Idea 5.

**TABLE 3:** First discussion excerpt of the low-performing group.

Turn	Speaker	Content	New Idea	Talk Move
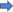 61	Bao	First, we know Ming has one more popsicle and ice cream than Lin. That is 8. So, one popsicle and one ice cream equal 8. So, back to the figure above, we can know Ming has 2 popsicles and 2 ice creams…	5	
62	Li	Ah?! Four popsicles		Disagree; Add on
63	Yan	Four popsicles (in soft voice)		
64	Xiao	I got it. I got it.		
65	Li	Four popsicles and 2 ice creams. Ha ha.		
66	Xiao	No, the 8 more yuan should be this…		Disagree
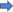 67	Li	The 8 more yuan is this and this. After subtracting these two:		Co-elaborate
68	Xiao	Two popsicles… (interrupt)		
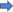 69	Li	After subtracting these two, there is one and three popsicles	6	
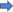 70	Bao	You two talked so much, which amounts to nothing!		Evaluate
71	Li	One ice cream and three popsicles.		
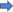 72	Xiao	No, this should be one group. One group costs 8. There are two groups. Two more left. The money left equals the price of two popsicles.	7	Disagree; Add on
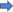 73	Li	That is exactly what I meant.		Agree
74	Xiao	Then, why did you say that again:		Why
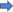 75	Bao	You two talked so much, which amounts to nothing! Ha ha, one ice cream and three popsicles:		Evaluate
76	Li	Next, please Xiao, expressed your idea. What you just said is quite good. You should talk to the recorder.		Invite to express

In addition to the ineffective positive feedback of “agree” for Idea 7, the disagreement concerning Idea 9 also failed to stop its growth. When Bao conducted specific calculations in Idea 9, he unfortunately made a mistake in calculating the price for one popsicle in turn 100 (see Table 4). However, his mistake received no immediate correction, as Xiao proposed to move on to the next item in turn 101. When Li referred back to Idea 9 after her mistake in reversing the prices of a popsicle and an ice cream in Idea 10 (turn 105), Xiao seemed to realize the problem with Idea 9 and expressed his disagreement with it (turn 106). However, his negative feedback was disregarded when Bao urged them to work on the next problem (turn 107).

**TABLE 4:** Second discussion excerpt of the low-performing group.

Turn	Speaker	Content	New Idea	Talk Move
98	Bao	Calculate 22 plus 14 first. This is the total money they spent that equals 36 Yuan. 36, then there are three groups of three ice creams and three popsicles. One group is 8 Yuan. 3 × 8 = 24, then there are four popsicles left. Then two, seven, three, eight (doing verbal arithmetic):	8	
99	Yan	Xiao’s solution is better (not very clear)		Evaluate
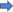 100	Bao	24 Yuan, 36, 36 minus 24 equals 12 Yuan. 36−24 = 12. 12 Yuan is the price for the four popsicles left. 12 dividing 4 equals 3 Yuan (4.0). So, we know every popsicle costs 4 Yuan. Then 3 × 4 = 12. Then the price for three popsicles is 12 Yuan. And that is 14 Yuan in total. 14−12 is the unit price of ice cream, that is 2 Yuan. The answer is that one ice cream costs 2 Yuan and one popsicle costs 4 Yuan.	9	Elaborate
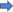 101	Xiao	The second problem, second problem (in soft voice).		
102	Li	Our problem has been finally solved. One popsicle costs 2 Yuan and one ice cream costs 4 Yuan.	10	
103	Bao	Ha ha, one ice cream costs 4 Yuan:		Disagree
104	Xiao	We are finished. One ice cream costs 4 Yuan. You recorded the opposite.		Disagree
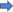 105	Li	I said that one popsicle cost 2 Yuan and then one ice cream. One ice cream cost 4 Yuan. One ice cream: Umh: one ice cream costs 2 Yuan and one popsicle costs 4 Yuan.		Elaborate
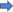 106	Xiao	That is still wrong. Something is wrong!		Disagree
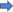 107	Bao	The second problem!		

#### The Idea Hierarchy Analysis

**FIGURE 7:**
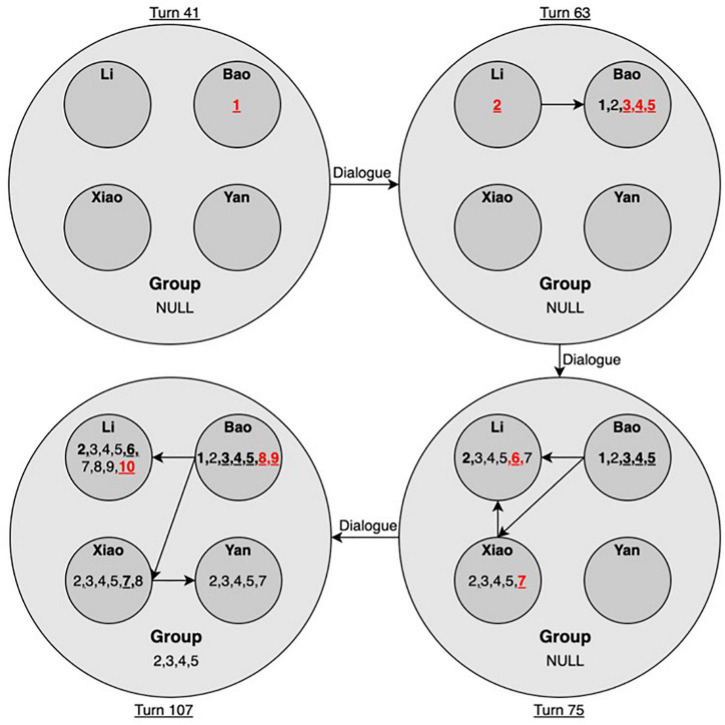
Idea hierarchy for the low-performing group (Note. Arrows denote the direction of idea flow. Numbers indicate the order of emergent ideas. Bold numbers with underlines indicate the idea was initially proposed by the indicated student. Numbers in red denote ideas that emerged in the current state).

We also selected four states of the idea hierarchy for the low-performing group to examine the dynamics of idea emergence at various levels. As shown in Figure 7, in the initial state at turn 41, the first idea emerged for Bao, who suggested drawing out the conditions. Li added onto Bao and put forward the second idea. Then, Bao added Idea 3. However, Xiao questioned Li and Bao, as they were not answering the question at turn 45. Li viewed Xiao as a very mathematically competent peer. She then shifted to ask Xiao and then Yan. However, both Xiao and Yan still did not have ideas. Then, Bao continued his thoughts and put forward Idea 5. Until turn 63, most ideas emerged for Bao. Only Li and Bao shared Idea 2, while the whole group had no explicitly shared ideas.

Turns from 63 to 75 were dominated by Li and Xiao. They competed for turns to build on Idea 5. Li added Idea 6, while Xiao added Idea 7. They achieved a consensus on Idea 7 at turn 73. However, their shared idea was not understood by Bao, nor vocally agreed to by Yan. Therefore, the group still did not vocally share any joint ideas until turn 75. From turn 76 to turn 94, Li and Xiao failed to work out the final answers based on Idea 7. Bao jumped in at turn 98 and proposed a new solution in Idea 8. Yan commented that this solution was not as good as Xiao’s Idea 7, which indicated she agreed with Xiao’s Idea 7 as well as the dependent ideas. Bao continued to work out the final answers in Idea 9 (turn 100). This idea was directly taken by Li and Xiao and later questioned by Xiao in the end (turn 107), while Yan did not vocally express her viewpoints toward Idea 9. Therefore, until turn 107, the final group answers (idea 9) were only shared by Li and Bao. In the group space, only Ideas 2, 3, 4, and 5 were explicitly shared by all members.

In brief, Bao proposed most of the ideas throughout the various states and mainly through intra dialogue, while Yan was a noticeably reticent participant. Li was an active contributor and a little bossy in their discussion. She shared most ideas with other peers as well. Xiao was viewed as the most competent in mathematics and proposed an essentially correct solution in Idea 7. As shown in Table 1, Xiao and Li had the highest mathematics grades and self-concepts, relative to Yan, who had the lowest mathematics grade and self-concept in the group. Bao’s mathematics grade was also lower than those of Li and Xiao. These status characteristics might explain why Li easily turned to Xiao for answers when her discussion with Bao was questioned by Xiao and why Yan was mainly silent in the discussion and did not contribute any new ideas. The dialogue in the low-performing group was constrained by obvious status problems, which prevented a sufficient flow of ideas among the group members.

## Discussion

### Discussion of the Study Findings

The present study found that new ideas emerged during D-CPS performed features of attractors in CDS in both high-performing and low-performing groups. The emergent new ideas attracted local utterances and evolved through multilevel and *trans*-level regulative loops. Talk moves of the evaluation type, in particular “agree” and “disagree,” helped control the bifurcations of an idea tree. This was consistent with Bloom (2001) early finding that arguments and counterarguments formed feedback loops to drive the argumentative talk forward and into complexity. However, the regulative impact of these evaluative talk moves in the low-performing group did not work as well as those in the high-performing group. It happened that “agree” failed to promote further development of the agreed idea, and “disagree” failed to end the present idea or shift the focus to a new one.

Attractors have different qualities (Kauffman, 1993). A strong attractor has a broad and deep basin of attraction that is more difficult for a system to escape. The quality of attractors can be changed through feedback loops and circular causality during interactions of substates of a system. This study showed that the basin of attraction for an emergent idea might get deeper and wider with constant positive feedback around it or shallower or narrower due to negative feedback. In addition to the evaluation of this idea, the speaker’s or evaluator’s status also tended to affect the quality of a certain idea attractor. Ideas contributed by a high-status student were more likely to have a large initial basin of attraction. Meanwhile, the influence of negative or positive feedback tended to be augmented by a high-status student.

### Implications of the Complex Dynamic Systems Perspective

The CDS perspective has both theoretical and methodological implications for research on learning interactions. It highlights the emergent and non-linear nature of learning and thus promotes an interaction-oriented research paradigm. The CDS theory resonates with the onto-epistemological assumptions of Bakhtinian dialogic theory. Many conceptual tools in CDS could therefore be borrowed to examine the dynamics of D-CPS from a fresh perspective. For example, the present study adopted the concept of attractor in CDS to examine idea emergence in D-CPS.

Attractors order the underlying complexity of a system (Boeing, 2016). One essential task of complex systems research is to expose the order that underlies apparently random phenomena (Patton, 1990). Attractors act as long-term and slow-changing order parameters and synergize all the other short-term and fast-changing parameters through constant feedback loops so that complex systems evolve and new system features emerge (Bateson, 1979; Haken, 1980). In the context of collaborative interactions, attractors provide a new perspective for understanding and interpreting the non-linear trajectories of student discussions.

Methodologically, the CDS perspective challenges the traditional reductionist approach as well as the temporal homogeneity underlying most statistical techniques. The large set of conceptual and methodological tools in CDS largely augment the temporal analysis of learning interaction (Koopmans and Stamovlasis, 2016; Kaplan and Garner, 2020).

There have been a lot of computational tools available to help detect attractors in learning interactions, such as the state space grid (Hollenstein, 2013) and recurrence quantification analysis (Belaire-Franch and Contreras, 2002). The present study proposed two visual qualitative tools, the diagrams of idea tree and idea hierarchy, to help researchers examine the trajectories of idea emergence in group discussions. The idea tree enabled an intuitive exploration of group-level idea evolution by illustrating the relationship between productive talk moves and emergent new ideas. It also allowed comparisons of trajectories of idea emergence across different groups or different problem-solving states of one group. A set of chronological idea hierarchy diagrams can further illustrate the dynamics of idea emergence across the individual, dyad, and group levels.

Visual and computational modeling have been widely used in systems science research to describe the dynamics of systems and to explore various possible states (e.g., Servedio et al., 2014). Visualizing time-series data helps viewers to intuitively see the structure of data and possible anomalies, clusters, or other regularities (Aghabozorgi et al., 2015). Increasing efforts have been made to develop visual learning analytics tools, not only to support group work and teacher guidance (e.g., Zhang et al., 2018; Van Leeuwen et al., 2019; Chen, 2020), but also to help researchers uncover the temporal patterns of D-CPS (e.g., Shaffer and Ruis, 2017; Lämsä et al., 2018; Borge and Mercier, 2019). CDS theory provides an alternative perspective to guide the design of such visual analytics tools and analyze the patterns of time-series conversation data.

### Limitations and Future Research Directions

There are some limitations of this study that call for future research. First, the empirical part could be strengthened to provide stronger support for the proposed theoretical framework. This study proposed a coherent theoretical framework based on CDS and Bakhtinian dialogism to guide the analysis of the dynamics of D-CPS. It further demonstrated how this framework guided the exploration of emergent ideas as possible attractors in group discussion by comparing the diagrams of idea tree and idea hierarchy between two selected groups. It remains open for future research to validate the current findings by analyzing the features of idea emergence in various groups and contexts. Future research could also adopt the proposed theoretical framework to explore other aspects of dynamics of group discussion to validate the usability of this framework.

Second, the generalizability of the findings could be enhanced. This study demonstrated the existence of attractors in D-CPS. New ideas in group discussion tended to attract local utterances. However, this was an exploratory study based on the qualitative comparisons of only two groups. More research is warranted to validate whether utterances containing new ideas were persistent attractors in group discussion outside the present setting. For example, different types of tasks may generate different styles of group discussion. This study focuses on reasoning tasks in mathematics which are typically featured by the sequencing of group talk (Stahl, 2014) and mainly require convergent thinking. Although discussion around creative tasks shares some common communicative patterns with that around reasoning tasks (Rojas-Drummond et al., 2006), it remains open for future research to examine whether the findings could generalize to problem solving that mainly requires divergent thinking. In addition, this study focuses on face-to-face discussion of foursomes in primary school. Future research could also explore whether the findings could generalize to other samples, other group sizes or online contexts.

This study yielded recommendations for how the diagrams of idea tree and idea hierarchy can help analyze the trajectories of idea emergence in dialogic group discussion. However, it was time consuming to manually draw these two diagrams that require fine-grained coding. This limited the overall scale of the analysis to only two groups in solving one mathematical problem. Prospective studies could further explore how to improve the production of these two diagrams or reveal other visual analytical approaches to analyze idea emergence in group discussion. It is also meaningful to explore other affordances of these two diagrams such as how the width and/or depth of the idea tree might relate to group performance or communicative patterns.

This study showed how the concept of attractor in CDS and a visual and qualitative approach could help explain the cognitive trajectories of D-CPS. It is also notable that Bakhtinian dialogic theory differs from CDS theory in its essential ethical considerations. D-CPS focuses on the interanimation of multiple independent voices but also the equal rights of these voices. Therefore, this study also implies that future research on D-CPS should consider whether there is any local participation hierarchy emergent in group discussion and the reasons behind it, which might necessitate an individual-level analysis.

## Conclusion

The present study conceptualized D-CPS as a CDS embedded in community and society. The introduced concept of attractor provides an alternative perspective for understanding and interpreting the trajectories of idea emergence in collaborative discussions. This study advocates CDS theory as an alternative perspective for examining the dynamics of D-CPS, given its ontological coalescence with Bakhtinian dialogic theory and its strong conceptual and methodological tools that could help unpack the complexity of learning interactions. Currently, the community of complexity theories in education is still small (Kaplan and Garner, 2020). We call for more efforts in applying CDS concepts and methods to education.

## Data Availability Statement

The raw data supporting the conclusions of this article will be made available by the authors, without undue reservation.

## Ethics Statement

The studies involving human participants were reviewed and approved by The University of Hong Kong Human Research Ethics Committee (HREC). Written informed consent to participate in this study was provided by the participants’ legal guardian/next of kin.

## Author Contributions

LH conceived the original idea and drafted the manuscript with support from GC. GC verified the analytical methods and helped supervise the project. Both authors contributed to the article and approved the submitted version.

## Conflict of Interest

The authors declare that the research was conducted in the absence of any commercial or financial relationships that could be construed as a potential conflict of interest.

## Publisher’s Note

All claims expressed in this article are solely those of the authors and do not necessarily represent those of their affiliated organizations, or those of the publisher, the editors and the reviewers. Any product that may be evaluated in this article, or claim that may be made by its manufacturer, is not guaranteed or endorsed by the publisher.

## Appendix

Ice **c**ream. Ming buys two ice creams and four popsicles. He spends 22 yuan in total. Lin buys one ice cream and three popsicles. She spends 14 yuan in total. How much do one ice cream and one popsicle cost? Please write out your problem-solving process in detail.

Answer: One ice cream costs ______ yuan.

One popsicle costs _____ yuan.

Your problem-solving process:
